# Prognostic score models for survival of nasopharyngeal carcinoma patients treated with intensity-modulated radiotherapy and chemotherapy

**DOI:** 10.18632/oncotarget.5781

**Published:** 2015-09-22

**Authors:** Lei Zeng, Pi Guo, Jin-Gao Li, Fei Han, Qiang Li, Yong Lu, Xiao-Wu Deng, Qing-Ying Zhang, Tai-Xiang Lu

**Affiliations:** ^1^ State Key Laboratory Oncology in South China, Collaborative Innovation Center of Cancer Medicine, PR China; ^2^ Department of Radiation Oncology, Sun Yat-Sen University Cancer Center, PR China; ^3^ Department of Radiation Oncology, Jiangxi Cancer Hospital, Nanchang, PR China; ^4^ Department of Medical Statistics and Epidemiology, School of Public Health, Sun Yat-Sen University, PR China; ^5^ Department of Preventive Medicine, Shantou University Medical College, Shantou, PR China

**Keywords:** nasopharyngeal carcinoma, prognostic score model, intensity-modulated radiotherapy, prognostic factors, nomogram

## Abstract

**Purpose:**

To establish accurate prognostic score models to predict survival for patients with nasopharyngeal carcinoma (NPC), treated with intensity-modulated radiotherapy (IMRT) and chemotherapy.

**Materials and methods:**

Six hundred and seventy-five patients with newly diagnosed, nonmetastatic and histologically proven NPC who were treated with IMRT and chemotherapy were analyzed retrospectively. Samples were split randomly into a training set (*n* = 338) and a test set (*n* = 337) to analyze. All data from the training set were used to perform an extensive survival analysis and to develop multivariate nomograms based on Cox regression. Data from the test set was used as an external validation set. Risk group stratification was proposed for the nomograms.

**Results:**

The nomograms are able to predict survival with a C-index for external validation of local recurrence-free survival (LRFS; 0.66, 95% CI: 0.58-0.74), distant metastasis-free survival (DMFS; 0.73, 95% CI: 0.66-0.79), and disease-specific survival (DSS; 0.73, 95% CI: 0.67-0.79). The calibration curve for probability of survival showed good agreement between prediction by nomogram and actual observation. The C-index of the nomogram for LRFS, DMFS and DSS were statistically higher than the C-index values of the AJCC seventh edition (*P* < 0.001). In the test set, the nomogram discrimination was also superior to the AJCC Staging systems (*P* < 0.001). The stratification in risk groups allows significant distinction between Kaplan-Meier curves for outcome.

**Conclusions:**

Prognostic score models were successfully established and validated to predict LRFS, DMFS, and DSS over a 5-year period after IMRT and chemotherapy, which will be useful for individual treatment.

## INTRODUCTION

The tumor-node-metastasis (TNM) staging system for nasopharyngeal carcinoma (NPC) plays an important role in predicting prognosis, facilitating treatment stratification, and exchanging experience among different treatment centers. However, TNM staging system may not be precise enough to predict prognosis of NPC because patients with the same TNM stage often have different prognoses. Various prognostic factors, such as lactate dehydrogenase (LDH) level in serum, body mass index (BMI), hemoglobin, neutrophil to lymphocyte ratio (NLR) and primary gross tumor volume (GTV-P), have also been identified and evaluated retrospectively [1-4].

In the past decades, the diagnostic and treatment methods for NPC have made great progress. Especially, intensity-modulated radiotherapy (IMRT) has improved local control and long-term survival for patients with NPC compared with two-dimensional conventional radiotherapy (2D-CRT) [5, 6]. Therefore, it is necessary to determine whether prognostic factors previously evaluated for 2D-CRT could also be applied to modern IMRT. More and more attention has been paid to individual treatment. That emphasizes the need to build a more accurate and practical prognostic system for predicting the clinical outcome of patients treated by IMRT and chemotherapy. The objective of this study is to establish a convenient prognostic score models for NPC patients treated with IMRT and chemotherapy.

## RESULTS

### Patterns of treatment failure and survival for the whole cohort

A total of 68 (10.1%) patients developed disease recurrence, 114 (16.9%) developed distant metastases and 136 (20.1%) died. A total of 136 patients died, among which 105 died of distant metastasis, 31 died of locoregional relapse, 3 died of other cancer, 2 died of intercurrent disease, 3 died of cardiovascular events, 2 died from car accident, and 4 died of unknown causes. The 5-year survival rates were as follows: local recurrence-free survival (LRFS), distant metastasis-free survival (DMFS), disease-free survival (DFS), disease-specific survival (DSS) and overall survival (OS) rates were 92.3%, 83.6%, 76.4%, 85.1% and 83.7%, respectively.

**Figure 1:**
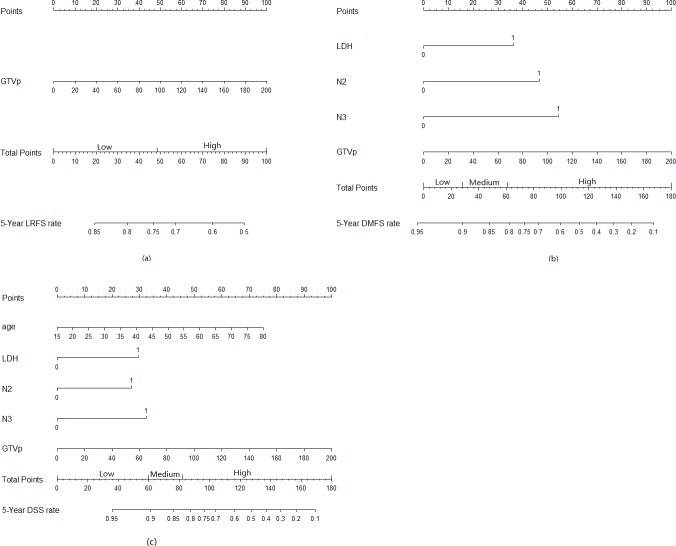
Nomograms developed for 5-year prediction of (a) local recurrence-free survival, (b) distant metastasis-free survival, (c) disease-specific survival

### LRFS, DMFS, DSS and independent prognostic factors in the training set

The median follow-up time was 70 months (range, 6 to 116 months). The 5-year LRFS, DMFS and DSS rates were 93.3%, 84.5% and 85.7%, respectively. The results of the Kaplan-Meier analyses are listed in Table 2. Multivariate analyses demonstrated that age, LDH, N2 classification, N3 classification and GTV-P have a significant impact on DSS time. GTV-P was the only one independent prognostic factor for LRFS. LDH, N2 classification, N3 classification and GTV-P affect significantly DMFS (Table 3).

**Table 1:** Characteristics of the 675 nasopharyngeal carcinoma patients

Characteristics	Training set (n=338)	Test set (n=337)
	No. of patients (%)	No. of patients (%)
Age (years)		
Median	43	43
Range	15-76	13-78
Gender		
Male	265(78.4%)	263(78.0%)
Female	73(21.6%)	74(22.0%)
Histology		
WHO type II	26(7.7%)	29(8.6%)
WHO type III	312(92.1%)	309(91.4%)
T classification		
T1	36(10.6%)	43(12.7%)
T2	75(22.2%)	82(24.3%)
T3	153(45.3%)	136(40.4%)
T4	74(21.9%)	76(22.6%)
N classification		
N0	43(12.7%)	43(12.8%)
N1	169(50.0%)	188(55.8%)
N2	93(27.5%)	80(23.7%)
N3	33(9.8%)	26(7.7%)
Clinical stage		
II	65(19.2%)	92(27.3%)
III	169(50.0%)	146(43.3%)
IVA-IVB	104(30.8%)	99(29.4%)

**Table 2:** LRFS, DMFS and DSS at 5 year for the training set, stratified for each variable

Factors	No. of patients (%)	LRFS (%)		DMFS (%)		DSS (%)	
		5 y	P	5y	P	5y	P
Age, years			0.059		0.210		0.011
<50	240(71.0%)	95.1		85.6		88.6	
≥50	98(29.0%)	88.8		81.7		78.4	
Gender			0.316		0.141		0.045
Male	265(78.4%)	92.7		83.4		83.7	
Female	73(21.6%)	95.6		88.7		92.9	
T classification		<0.001			0.066		<0.001
T1	36(10.6%)	100.0		91.7		94.4	
T2	75(22.2%)	100.0		89.2		91.8	
T3	153(45.3%)	93.6		84.6		85.1	
T4	74(21.9%)	82.7		75.4		76.5	
N classification			0.943	<0.001			0.001
N0	43(12.7%)	93.0		100.0		93.0	
N1	169(50.0%)	94.4		89.5		90.8	
N2	93(27.5%)	91.6		73.9		78.2	
N3	33(9.8%)	92.9		69.1		71.9	
Anemia before treatment			0.049		0.742		0.207
Yes	29(8.6%)	84.4		81.5		77.4	
No	309(91.4%)	94.1		84.8		86.4	
NLR before treatment*			0.090		0.607		0.052
≤1.63	85(25.1%)	93.5		86.6		87.7	
∼2.23	85(25.1%)	97.5		88.1		91.6	
∼3.00	85(25.1%)	95.1		83.2		85.5	
>3.00	83(24.7%)	87.0		79.9		77.8	
LDH before treament†			0.232	<0.001			<0.001
≤245 IU/L	310(91.7%)	93.8		86.4		87.4	
>245 IU/L	28(8.3%)	87.7		62.7		67.3	
GTV-P¶		<0.001			0.167		0.005
≤15.1 ml	84(24.9%)	98.8		91.7		94.0	
∼25.4ml	85(25.1%)	96.0		85.6		85.3	
∼48.5ml	85(25.1%)	94.9		82.2		88.2	
>48.5 ml	84(24.9%)	83.4		78.1		75.1	
Cigarette smoking			0.096		0.919		0.662
Yes	140(41.4%)	93.4		84.2		84.3	
No	198(58.6%)	93.2		85.1		86.7	
Chemotherapy			0.457		0.129		0.177
CCT‡	184(44.0%)	94.2		87.9		88.3	
Neoadjuvant+CCT	138(50.4%)	93.0		79.7		82.8	
CCT+adjuvant	16(5.6%)	86.5		86.5		80.8	

* Categorized into 4 groups according to quartile.

† Normal LDH level: 109.0-245.0 IU/L.

¶ Categorized into 4 groups according to quartile.

‖ Categorized into 4 groups according to the World Health Organization (WHO) classification for Asian populations.

‡ Concomitant chemotherapy.

**Table 3:** Multivariate analyses to determine the final predictors for the nomograms

Factors	Cox proportional hazards regression			nomogram		
	HR	95% CI	P	Performance (c-index)	95% CI
LFRS				Training:0.65	0.58-0.74
GTV	1.018	1.010-1.025	<0.001	Test:0.66	0.56-0.73
DMFS					
LDH	2.227	1.017-4.875	0.045		
N2	2.828	1.577-5.072	<0.001	Training:0.76	0.65-0.86
N3	3.369	1.465-7.748	0.004	Test:0.73	0.66-0.79
GTV	1.011	1.005-1.018	0.001		
DSS					
Age	1.033	1.011-1.056	0.003		
LDH	2.326	1.044-5.179	0.039	Training:0.76	0.70-0.81
N2	2.178	1.236-3.840	0.007	Test:0.73	0.68-0.78
N3	2.523	1.068-5.963	0.035		
GTV	1.014	1.009-1.020	<0.001		

Note. The concordance index (c-index; mean and 95% CI) for the training and test sets are given for the derived nomograms as a performance measure.

Abbreviations: HR: hazard ratio.

CI: confidence interval. Age and GTV are continuous.

### Prognostic nomogram for LRFS, DMFS and DSS

**Figure 2:**
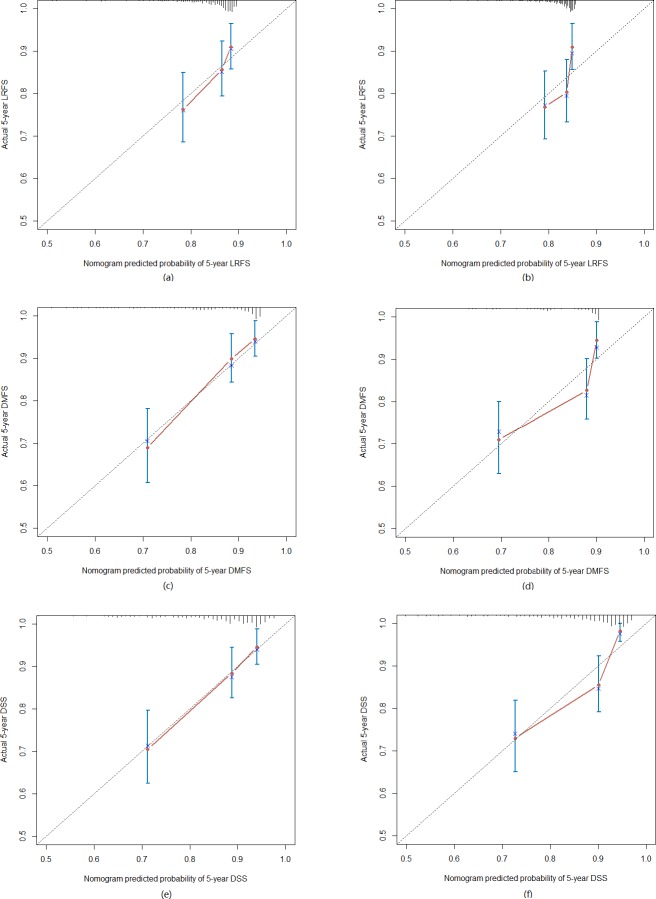
The calibration curve for predicting patient 5-year survival at (a) LRFS in the training set, (b) LRFS in the test set; (c) DMFS in the training set, (d) DMFS in the test set; (e) DSS in the training set, (f) DSS in the test set

The nomogram that combined all significant independent prognostic factors for LRFS, DMFS and DSS in the training set is shown in Figure 1. The calibration plot for the probability of survival at 5-year after treatment revealed a good match between the prediction by nomogram and actual observation (Figure 2).

### Comparison of predictive accuracy for LRFS, DMFS and DSS between nomogram and the AJCC staging systems

The nomogram showed better accuracy in predicting LRFS, DMFS and DSS in the training set. The C-index of the nomogram for LRFS was much higher than that of T stage of the 7th edition of the AJCC staging system (0.65, 95% CI: 0.58-0.74 *vs*. 0.60, 95% CI: 0.54-0.66, *P* < 0.001). The C-index of the nomogram for DMFS was much higher than that of N stage of the 7th edition of the AJCC staging system (0.76, 95% CI: 0.65-0.86 *vs*. 0.70, 95% CI: 0.61-0.80, *P* < 0.001). The C-index of the nomogram for DSS was much higher than that of the 7th edition of the AJCC staging system (0.76, 95% CI: 0.70-0.81 *vs*. 0.66, 95% CI: 0.60-0.73, *P* < 0.001).

### Validation of predictive accuracy of the nomogram for LRFS, DMFS and DSS

LRFS prediction performed with a C-index of 0.66 (95% CI: 0.56-0.73). For DMFS, the final model's C-index was 0.73 (95%CI: 0.66-0.79). The nomogram for DSS had a C-index of 0.73 (95%CI: 0.68-0.78). These validation performances are not significantly lower than the performances on the training set, which makes overfitting by the model less evident. A calibration curve showed good agreement between prediction and observation in the probability of 5-year survival (Figure 2). The C-index of the AJCC seventh edition for LRFS was significantly lower than that of the nomogram (0.59, 95% CI: 0.52-0.66, *P* < 0.001). The C-index of the AJCC seventh edition for DMFS was significantly lower than that of the nomogram (0.67, 95% CI: 0.59-0.73, *P* < 0.001). The C-index of the AJCC seventh edition for DSS was significantly lower than that of the nomogram (0.65, 95% CI: 0.59-0.72, *P* < 0.001).

### Subgroups according to quartiles of the risk score in the training set and test set

**Figure 3:**
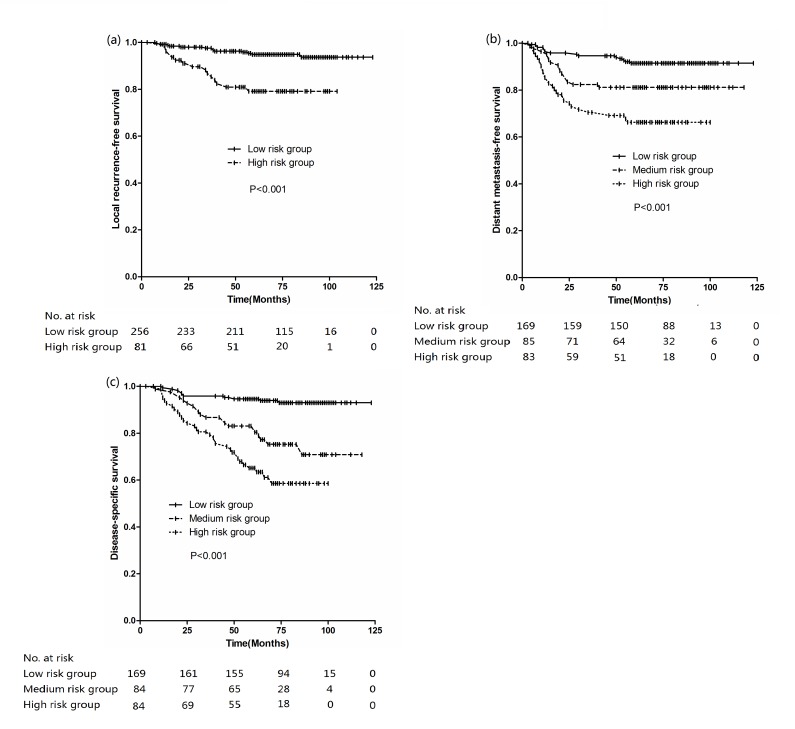
Kaplan-Meier curves stratified by different risk group (a) LRFS, (b) DMFS, (c) DSS

By splitting the training set into four subgroups with different quartiles of the risk score for LRFS, we have identified a high- and low-risk group. No differences, however, were found among the three groups; thus, we merged these groups on patient group with a low risk score. Kaplan-Meier estimates of the LRFS rate for the test set displayed statistically different outcomes for the two proposed risk groups (5-year LRFS: 94.9% for low risk group; 79.2% for high risk group; *P* < 0.001, Figure 3a).

By splitting the training set into four subgroups with different quartiles of the risk score for DMFS, we have identified a high- and low-risk group. No differences, however, were found between the other two groups; thus, we merged these groups on patient group with a low risk score. Kaplan-Meier estimates of the DMFS rate for the test set displayed statistically different outcomes for the three proposed risk groups (5-year DMFS: 91.4% for low risk group; 81.1% for medium risk group; 66.3% for high risk group; *P* < 0.001, Figure 3b).

By splitting the training set into four subgroups with different quartiles of the risk score for DSS, we have identified a high- and low-risk group. No differences, however, were found between the other two groups; thus, we merged these groups on patient group with a low risk score. Kaplan-Meier estimates of the DSS rate for the test set displayed statistically different outcomes for the three proposed risk groups (5-year DSS: 94.6% for low risk group; 83.1% for medium risk group; 65.1% for high risk group; *P* < 0.001, Figure 3c).

## DISCUSSION

Recently, the application of IMRT and combination of chemotherapy and radiotherapy have significantly improved long-term survival for NPC patients. Thus, it is of interest to reevaluate the previous existing prognostic factors for NPC in the new era of IMRT.

### LRFS prediction

The aim of this study was to develop a nomogram for LRFS to predict LRFS and select patients for neoadjuvant chemotherapy to improve local control. The best model for LRFS was only based on GTV-P.

A retrospective study by Sze et al. [10] suggested that GTV-P was an independent prognostic factor for the local failure-rate, and the risk of local failure was estimated to increase by 1% for every 1 cm^3^ increase in primary tumor volume. Guo et al. [11] reported that only GTV-P was an independent prognostic indicator for LFRS and significantly improves the prognostic validity of T classifications in NPC. Wu et al. [12] analyzed the correlation between GTV-P and prognosis in patients with NPC undergoing IMRT. Multivariate analysis showed that only GTV-P had a statistically significant correlation to local control, whereas T classification was not associated with LRFS. It concurred closely with our findings that not T classification but GTV-P significantly had significant effect on 5-year LRFS in the current study. This indicates the limitations of the current T staging system, which is based on anatomic location and cranial nerve involvement and cannot accurately reflect the tumor bulk.

Benefit of neoadjuvant chemotherapy has been seen in reduction of local failure [13]. The results from two meta-analysis also showed that the addition of neoadjuvant chemotherapy (NACT) to radiation significantly reduced the incidence of locoregional recurrences, and no significant beneficial effect on the incidence of locoregional recurrence was found for adjuvant chemotherapy (ACT) [14, 15]. However, no significant differences in 5-year LRFS were observed among the three groups (concurrent chemotherapy (CCT), CCT+NACT, CCT+ACT) in the current series (Table 2). One reasonable explanation is that the patients with advanced disease were more likely to receive NACT. So, we can select patients for NACT based on GTV-P. Although the estimated probabilities by the nomograms are on a continuous scale, we proposed two risk groups of local recurrence based on GTV-P which stratified by 48.5 ml(5-year LRFS: 94.9% for low risk group; 79.2% for high risk group; *P* < 0.001, Figure 3a). In agreement with our findings, Wu et al reported that 48 ml was the cutoff point of GTV-P for local control, and the 5-year local recurrence-free for patient with GTV-P smaller than 48 ml and greater than 48ml were 98.0% and 77.9%, respectively (*P* < 0.001). So, different treatment strategies could be followed for the two categories (CCT, NACT+CCT).

### DMFS prediction

With the use of IMRT, coupled with the wide adoption of concomitant chemotherapy, the local relapse rate in NPC has significantly decreased, and distant metastases has become to be the predominant model of treatment failures [6], the identification and stratification of patients at high risk for metastasis could optimize staging and treatment strategies.

The aim of this study was to develop a nomogram to predict metastasis rates and select patients for neoadjuvant chemotherapy. The best model for distant metastasis resulted in the following factors: LDH, N2 classification, N3 classification and GTV-P.

Up-regulation of LDH ensures an efficient anaerobic/glycolytic metabolism while enabling tumor cells to become independent of an oxygen supply. Meanwhile, high serum LDH level may reflect a large tumor burden and high probability of developing clones resistant to treatment. Consistent with our study, Zhou et al reported that serum LDH level before treatment was an adverse prognostic factor for distant metastasis [16]. A retrospective study reported by Wan et al showed that high pretreatment LDH value was also associated with an inferior 5-year DMFS [17].

Large tumor volumes usually indicate a high proliferation rate and malignancy of tumor cells, which has correlation to distant metastasis. Here, we confirmed that GTV-P affected significantly DMFS. Consistent to our present study, previous studies have demonstrated that GTV-P had a significant effect on distant metastasis of NPC [11, 12].

Two meta-analysis indicated that neoadjuvant chemotherapy had a significant impact on reduction of distant metastasis, and adjuvant chemotherapy had limited positive effect on distant metastasis [14, 15]. In contrast, the combination of neoadjuvant/adjuvant chemotherapy (cisplatin plus 5-fluorouracil regimen) and concurrent chemotherapy had modest effect on the risk of distant metastasis compared with concurrent chemotherapy alone (Table 2) in our study. The main reason may be that it was more likely for patients with advanced disease to receive neoadjuvant/adjuvant chemotherapy. The nomograms can serve as a guide for clinicians to choose neoadjuvant chemotherapy considering the risk for distant metastasis. Although all the patients from the medium risk and high risk were treated with chemoradiotherapy, they still had a high incidence of distant metastasis. Thus, the chemotherapy strategies we have fall short as treatments for these patients and, with better treatment outcomes in mind, we need more studies in order to find a comprehensive approach that works more intensively. Among other newer agents, taxanes and gemcitabine have demonstrated results that are promising with neoadjuvant and palliative chemotherapy in NPC [18, 19]. For the treatment of advanced NPC, Hui et al did a randomized phase II trial on the concurrent cisplatin radiotherapy with neoadjuvant docetaxel and cisplatin or without [20]. The preliminary results demonstrate that neoadjuvant chemotherapy shows its potential by reducing the distant metastasis. As a result, newer practice to combine more tolerable drugs which is likely to enhance the efficiency of chemotherapy as an adjunct in medium risk and high risk patients should be investigated. Moreover, the addition of molecular targeted agents to chemoradiotherapy may provide a survival benefit for these patients by eradicating micro-metastases [21, 22].

### DSS prediction

Disease-specific survival is more dependent on the DFMS than LFRS. The most predictive model for DSS was based on predictors similar to those for DFMS: age, LDH, N2 classification, N3 classification and GTV-P.

Treatment responses and compliance to treatment seem to change with age: elderly patients displayed a worse DSS than younger patients in the present study. Consistent to our study, Zhou et al.'s [16] and Chang et al.'s [3] studies revealed that there was a significant difference in survival between patients younger than 50 years and those older than 50 years.

Consistent to our present study, several groups have reported that serum LDH level before treatment was an adverse prognostic factor in NPC [1, 16, 17]. Chen et al. [23] and Guo et al. [11] reported that GTV-P was an independent prognostic indicator for overall survival. Wu et al’ study also indicated that GTV-P had a statistically significant correlation to overall survival. The previous studies concurred closely with our findings that GTV-P was associated with DSS in the current study.

Nomograms have been developed and shown to be more accurate than the conventional staging systems for predicting prognosis in some cancers [9, 24]. The nomograms performed well in predicting survival, and its prediction was supported by the C-index and the calibration curve. When compared with the 7th edition of the AJCC staging systems, the nomogram displayed better predictive accuracy for DSS in our study.

A prognostic index derived by combining points for each of these characteristics included age, LDH, N2 classification, N3 classification and GTV-P accurately separated patients into categories at the low risk, medium risk and high risk for disease-specific death. Different treatment strategies could be followed for each of these categories (radiotherapy alone, concurrent chemotherapy, and neoadjuvant/adjuvant+concurrent chemotherapy) and tailored schedule could be randomly tested any category to test the value of multidrug schedules.

### The limitations of our study

This study had several limitations. First, the nomograms were developed based on data obtained from a single institution in China. Second, although the prognostic value of pretreatment and post-treatment plasma Epstein-Barr viral (EBV) DNA levels has been validated in non-metastatic NPC patients [25, 26], only 15.3% (103/675) patients had plasma EBV DNA copy number before treatments in this series. It is not available for us to take EBV DNA into the model analysis. Further research is merit in future. Third, because no patient had histology of WHO I type, the model should be applied with caution in NPC patients with WHO type I disease.

## CONCLUSIONS

Prognostic score models were successfully established and validated to predict LRFS, DMFS, and DSS over a 5-year period after IMRT and chemotherapy, which will be useful for individual treatment.

## MATERIALS AND METHODS

### Patient characteristic

A total of 675 patients with newly-diagnosed, nonmetastatic and histologically proven NPC were treated with IMRT and chemotherapy in our Center from January 2003 to October 2008. The 675 NPC patients were randomly split into a training set (*n* = 338) and a test set (*n* = 337) by software with ratio of 1:1. A programmer (Guo Pi), who was blind to the clinical information before data analyses, performed the computerized randomization using randomization sequence. The characteristics of patients in training set and test set are shown in Table 1. Our study was approved by the ethics committee of our Cancer Center.

### Clinical staging

All patients completed pretreatment evaluations that included physical examination, hematologic and biochemistry test, fiberoptic endoscope examination of the nasopharynx, magnetic resonance imaging of the neck and nasopharynx, chest radiograph, bone scintigraph, and ultrasonography of the abdominal region. All NPC cases were restaged according to the 7th edition of the AJCC staging system.

### Treatment methods

#### Radiotherapy

Target volumes were delineated according to our institutional treatment protocol, in agreement with the International Commission on Radiation Units and Measurements Reports 50 and 62 [7-8]. Planning target volumes (PTVs) of GTVs and CTVs were generated automatically by adding a 3-mm margin after delineation of tumor targets according to the immobilization and localization uncertainties. Inverse planning was performed on the Corvus System for all patients using Simultaneous Modulated Accelerated Radiation Therapy boost RT. The prescribed dose was 68Gy to the PTV of the GTVnx, 60Gy to the PTV of CTV1 (i.e., high-risk regions), 54Gy to the PTV of CTV2 (i.e., low-risk regions), and 60-66Gy to the PTV of the GTVnd for the positive cervical lymph nodes in 30 fractions.

#### Chemotherapy

Of the 675 patients, 377 (55.9%) patients received concomitant chemotherapy. A combination of neoadjuvant and concomitant chemotherapy was delivered to 267(39.6%) patients, and concomitant and adjuvant chemotherapy were delivered to 31 (4.5%) patients. Neoadjuvant or adjuvant chemotherapy consisted of cisplatin (80mg/m^2^ IV on day 1) with 5-fluorouracil (800mg/m^2^ continuously IV on day 1-5) every three weeks for two or three cycles. Concurrent chemotherapy consisted of cisplatin (80mg/m^2^ IV) given on weeks 1, 4 and 7 of radiotherapy.

#### Follow-up and statistical analysis

The follow-up duration was calculated from the first day of therapy to either the day of death or the day of the last examination. After the completion of radiotherapy, all patients were followed up every 1-3months during the first 2 years, every 6 months in years 2 to 5 and annually thereafter. The median follow-up period was 70 months (range: 3-123 months).

All analyses were performed with SPSS software, version 19.0. Survival curves were depicted using the Kaplan-Meier method and compared using the log-rank test. Multivariate analyses with the Cox proportional hazards model were used to test for independent significance parameters by backward elimination. A nomogram was formulated based on the results of multivariate analysis and by using the package of rms in R version 2.10.1. A final model selection was performed by a forward step-down selection process with the Akaike information criterion. The performance of the nomogram was measured by concordance index (C-index) and assessed by comparing nomogram-predicted versus observed Kaplan-Meier estimates of survival probability. Comparison between the nomogram and the seventh edition of the AJCC staging system was performed with the rcorr.cens package in Hmisc in R and were evaluated by the C-index. The larger the C-index, the more accurate was the prognostic prediction [9]. During the external validation of the nomogram, the total points of each patient in the test set were calculated according to the established nomogram, then Cox regression in this set was performed using the total points as a factor, and finally, the C-index and calibration curve were derived based on the regression analysis. The training set and test set was split into four subgroups according to quartiles of the risk score. To assess for differences in survival of the subgroups Kaplan-Meier curves were made. *P* < 0.05 was considered statistically significant.
